# Surgical ‘Safari’ *vs.* Educational Program: Experience with International Cardiac Surgery Missions in Nigeria - A Rejoinder

**DOI:** 10.21470/1678-9741-2020-0694

**Published:** 2021

**Authors:** Okonta KE, Falase Bode, Adamu YB, Olugbemi AJ, Aminu MB, Onakpoya UU

**Affiliations:** 1 University of Port Harcourt, Rivers State, Nigeria; 3 Lagos State University Teaching Hospital, Lagos State, Nigeria; 4 Federal Medical Centre Keffi, Nasarawa State, Nigeria; 5 Tristate Cardiovascular, Babcock University Teaching Hospital, Ogun State, Nigeria; 6 Ahmadu Bello University Teaching Hospital, Kaduna State, Nigeria; 7 Obafemi Awolowo University Teaching Hospital, Ile-Ife, Osun State, Nigeria

Dear Editor,

The Association of Cardiovascular and Thoracic Surgeons of Nigeria (ACTSON), the umbrella body for practicing cardiovascular and thoracic surgeons in Nigeria, wishes to respond to an article authored by Nwafor et al.^[1]^ and published in the Brazilian Journal of Cardiovascular Surgery wherein the cardiac surgical practice in Nigeria was depicted as an abysmal failure and completely dependent on surgical safaris.

While we agree that cardiac surgical practice in Nigeria is not yet at the level of developed countries, we wish to state that over the past decade there has been a lot of progress with open-heart surgery programs being started in several centres^[2,3]^.

Many developed countries have surgical mission programs to help assist in patient management in developing countries^[4]^. This has helped to bridge the gaps created by lack of sufficient equipment, funding, and manpower. The main advantage of cardiac surgical missions is to assist in these areas and not to be responsible for the training of doctors and other surgical personnel. The discussion in this publication authored by Nwafor et al. as to whether adequate training was provided by the mission programs is not appropriate. The other reason for cardiac surgical missions is to assist the local surgeons to improve on their practice and keep them motivated to keep pushing in spite of the gross dearth of equipment and facilities in their environment. Cardiac surgical missions also help with skills transfer and development of confidence by the local team, as was the case with the programs of the Tristate Group, Obafemi Awolowo University Teaching Hospital (OAUTH) and The Lagos State University Teaching Hospital (LASUTH), which all had the support of cardiac surgical missions but have now transitioned to independent surgical programs by resident teams.

These institutions have performed 279 surgeries in the last three years, all by the indigenous surgeons^[5]^. It is therefore inaccurate to describe these programs as having “abysmally failed”, as alluded to by Nwafor et al.

Another inaccuracy in that publication is the open-heart surgery activity attributed to Gwagwalada Teaching Hospital, Abuja. This hospital has never had a cardiac surgery program.

In conclusion, contrary to the inaccuracies in the publication authored by Nwafor et al., many hospitals in Nigeria have transitioned to indigenous open-heart surgery with the support of various cardiac surgical missions and we still welcome the assistance provided by cardiac mission groups to help bridge the gap in tackling cardiac diseases in Nigeria.

## References

[1] Nwafor IA, Vickram A, Osenmobor KO (2020). Surgical “safari” vs. educational program: experience with international cardiac surgery missions in Nigeria. Braz J Cardiovasc Surg.

[2] Falase B, Sanusi M, Animasahun A, Mgbajah O, Majekodunmi A, Nzewi O (2016). The challenges of cardiothoracic surgery practice in Nigeria: a 12 years institutional experience. Cardiovasc Diagn Ther.

[3] Okonta KE, Tobin-West CI (2016). Challenges with the establishment of congenital cardiac surgery centers in Nigeria: survey of cardiothoracic surgeons and residents. J Surg Res.

[4] VOOM Foundation [@VOOMFdn] Congrats to Dr. Bode Falase & Dr. Reza Khodaverdian for FIVE successful surgeries! VOOM has concluded the Lagos mission and is headed to Enugu to the University of Nigeria Teaching Hospital for the second part of our mission!.

[5] (2020). Nigeria Heart Registry.

